# The Voluntary Hospital Essential to Public Safety

**Published:** 1919-09-13

**Authors:** 


### THE VOLUNTARY HOSPITAL ESSENTIAL TO PUBLIC SAFETY.

### I. Its Position of Financial Strength.

In recent days there have been several proposals relating to the provision of hospitals for the use of the British public. These projects and proposals from their nature seem to indicate that their source is tainted with political propaganda, and with the desire, intended or unconscious, to form public opinion in favour of new establishments, which are to be given up largely to science, including research. In these circumstances THE HOSPITAL has a grave responsibility for the policy advocated in its columns. This policy, from the birth of this journal nearly thirty-five years ago, has been the upholding, extension, and support of the voluntary system of hospitals throughout the United Kingdom. That system owes its popularity and strength, which is greater than that of any other hospital system the world has ever known, to the fact that the one principle which dominates the conduct of these institutions is, first and paramount, the best interests and welfare of the patients who are fortunate enough to obtain admission within the walls of a voluntary hospital of the best.

If we may judge by the utterances attributed to the weaker members attached to institutions within the voluntary field that are finding a place in the Press day by day during the off-season of the newspaper world, the financial condition of the voluntary hospitals is so acute that they are threatened with bankruptcy, and nothing can save them but the modern and weak panacea of feeble folk—namely, Government money and Government control. This dangerous and panicky plea is unworthy of the administration of any great voluntary hospital, whose managers must have, in the pecuniary results exhibited by their finance over a period of years, abundant grounds to thank God and take courage that under vigorous business management the voluntary system provides a fertile field for reaping the richest harvest, when it rests on past records and the work it has accomplished for all classes of the people. The faint hearts and the least stable members of the hospital firmament, who lack robustness and the accumulated strength of intelligent experience and well-doing of the best, have a habit of wringing their hands in public and intimating by suggestion, lacking the courage for terse statement, that their hospitals at any rate are rapidly approaching a state of bankruptcy which must surely come, as night follows day, unless Government aid is immediately forthcoming.

There are a number of people, and a few of them may be found in the hospital field, who delight in forecasts of failure and misery, which makes us a little sorry, though it is a national duty, to dissipate these vapourings and forebodings of evil to hospital finance. We have not space here to deal at length with this aspect of a great question, but, despite the claim of the miserable that figures can be made to prove anything, we have the privilege to demonstrate by audited figures the actual facts and position to-day of upwards of two thousand great charities, including those of 846 hospitals.

### THE SURPLUS INCOME OF 2,002 CHARITIES.

Taking the charities first, we find that for the year ending December 31, 1917, 2,002 had an income from all sources of £16,709,000, of which £14,266,000 came from subscriptions and donations, invested funds, receipts from patients, and contributions for general purposes; £1,560,000 from legacies; and £883,000 from donations given towards building funds and special purposes. These figures show an increase of income in 1917 amounting to £2,342,000 compared with that received in 1916. In the last five years the charities as a whole have received more each year than they have expended. Again the surplus income has amounted in each of these five years to the following sums:—

| | |
|---|---|
| In 1913 the surplus income amounted to | £1,278,000 |
| „ 1914 „ „ „ | 764,000 |
| „ 1915 „ „ „ | 912,000 |
| „ 1916 „ „ „ | 1,369,000 |
| „ 1917 „ „ „ | 1,486,000 |

Thus the total excess of income received by the charities during the five years ending 1917 was no less than £5,809,000. It will be seen, therefore, despite all the competition of war funds, all the heavy and growing taxation, and other and increasing claims of various kinds, the voluntary system has steadily extended its influence as a money-raiser from British people year by year.

### THE SURPLUS INCOME OF 846 HOSPITALS.

Passing now from the general to the particular, let us examine the audited figures of 846 hospitals. These hospitals are situated all over the United Kingdom—*i.e.*, in London, the Provinces, Scotland, and Ireland. The surplus income of these 846 voluntary hospitals amounted in each of the five years 1913-1917 to the following sums:—

| | |
|---|---|
| In 1913 the surplus income amounted to | £428,000 |
| „ 1914 „ „ „ | 276,000 |
| „ 1915 „ „ „ | 326,000 |
| „ 1916 „ „ „ | 703,000 |
| „ 1917 „ „ „ | 737,000 |

This gives a total surplus income in five years in the 846 hospitals of £2,470,000. Of course, some of the 846 hospitals had much larger surpluses than others. These figures demonstrate the remarkable facts (*a*) that the voluntary system as it at present works yields every year a large surplus of total

**The Voluntary Hospital Essential**—(*continued*).

income over total expenditure, and (*b*) that taking the whole of the 846 hospitals included in these calculations the voluntary system in the five years 1913-1917—that is, in the war years, the most trying years financially which hospitals have ever experienced—provided an average surplus income for each of the 846 hospitals of between £500 and £600. It is not yet possible to give the audited figures for 1918, but we have reason for the forecast that they are likely to show a greater surplus income for 1918 than the large sum of £737,000 in 1917.

Oh, ye hospital croakers and lay journalists out for sensation! Will you, in justice to yourselves and in mercy to British people of all classes, who owe so much to the voluntary hospital system, absorb the truths which underlie our statements and figures, shed your present methods, and put your strength and faith in the greatest and most important hospital system the world has ever possessed? Whether this be so or not, we hope to have the pleasure of presenting you with a yet further and more important and appetising menu in the next issue of THE HOSPITAL.

